# A novel approach for inventory problem in the pharmaceutical supply chain

**DOI:** 10.1186/s40199-016-0144-y

**Published:** 2016-02-24

**Authors:** Gökçe Candan, Harun Reşit Yazgan

**Affiliations:** Industrial Engineering Department, Sakarya University, 54187 Sakarya, Turkey

**Keywords:** Pharmaceutical supply chain, Shelf life, MILP, Vendor managed inventory

## Abstract

**Background:**

In pharmaceutical enterprises, keeping up with global market conditions is possible with properly selected supply chain management policies. Generally; demand-driven classical supply chain model is used in the pharmaceutical industry. In this study, a new mathematical model is developed to solve an inventory problem in the pharmaceutical supply chain.

**Method:**

Unlike the studies in literature, the “shelf life and product transition times” constraints are considered, simultaneously, first time in the pharmaceutical production inventory problem. The problem is formulated as a mixed-integer linear programming (MILP) model with a hybrid time representation. The objective is to maximize total net profit. Effectiveness of the proposed model is illustrated considering a classical and a vendor managed inventory (VMI) supply chain on an experimental study.

**Results:**

To show the effectiveness of the model, an experimental study is performed; which contains 2 different supply chain policy (Classical and VMI), 24 and 30 months planning horizon, 10 and 15 different cephalosporin products. Finally the mathematical model is compared to another model in literature and the results show that proposed model is superior.

**Conclusion:**

This study suggest a novel approach for solving pharmaceutical inventory problem. The developed model is maximizing total net profit while determining optimal production plan under shelf life and product transition constraints in the pharmaceutical industry. And we believe that the proposed model is much more closed to real life unlike the other studies in literature.

## Background

Pharmaceutical industry applies a supply chain policy that allows the continuation of a wide variety of materials with large quantities in a very fast flow. Within pharmaceutical supply chains, the product variety is a huge problem to manage within short time windows. Nevertheless, depending on the medicine drugs, amounts can be a big problem to trade with costs. In here, the requirements of small batches are particularly hard to handle. The production of pharmaceutical products has two stages as primary and secondary level. Primary production includes the production of basic molecules active components or pharmaceutical active ingredients. The secondary production also includes the processes of being formulated of these active components and the delivery to the customers. Many operations in pharmaceutical production occur in bulks called charge. Quality control also takes place with monitoring each charge. On the production line, cleaning is a matter in the case of product change (transition) and this situation is to prevent the contamination of different products. Besides, raw materials and products have a certain shelf life. All these constraints are reducing the efficiency in the pharmaceutical industry. Shelf life controls are performed for raw materials by subjecting to retest procedure at certain intervals. During retest, raw materials are kept in quarantine; they are not definitely included in the production line and if test results indicate that the raw materials can already be used, they are taken from the quarantine and transferred to production stores. In shelf life control for the products, expiration date is printed on the packaging while the product is on production line and the expiry date starts from the date of production.

Compared with supply chain of other products, pharmaceutical supply chain is very complex. The factors such as long set-up times, resource-intensive operations, short shelf life and high production of waste make the pharmaceutical supply chain different from other sectors. Pharmaceutical production is demand managed. Firms rarely deliver the product to pharmacy or patient; instead of this, they deliver products to the consumer through wholesalers (pharmaceutical warehouses).

In such a different featured sector to maintain a presence in the market despite all these constraints is possible with correctly selected supply chain management policies. To adapt to changing market conditions, sustainable supply chain policy and to compete in global market, the pharmaceutical supply chain should be carried out by mathematical models based on scientific formulas determined with correct strategies [27]. In planning, the importance of inventory management has also great importance. Considering countless complications, it is very difficult to obtain optimal schedules. However, mathematical models help to take right decisions.

For an optimal production plan in the pharmaceutical industry, cleaning and preparation times (these occur on product transition times), facility maintenance times, testing and the production of new chemicals, resource allocations, manpower utilization and inventory management must be decided and planned in an integrated way. This case requires a production planning strategy evaluating operational configurations with repeated consultations with several departments, process constraints, statistical combinations and business scenarios. The production must be planned at certain time intervals and in accordance with a hierarchical approach [11]. In planning, the importance of inventory management has also great importance. Also to compete in pharmaceutical production in the global market, it is required to develop effective inventory control policies. Companies want to meet customer demands at the highest levels and prefer product storage to avoid falling below a safety stock level. In this way, high amount of inventory cost occurs. While reducing the inventory level for minimizing costs, firms cannot meet the demands, delivery dates delays, and there are some decreases in service levels [30].

With the latest developments in information technologies, the fast and easy internet networks are used to make the information sharing easier and increase security across the supply network. Accordingly, VMI defined as “cooperation with a customer and a supplier to optimize an inventory management for least-cost on both companies” began to be used. With this model, the supplier takes responsibility for the operational management of inventory with agreed performance targets. These performance targets are continuously monitored and updated to ensure continuous improvement [12]. In order to optimize supply chain performance, the manufacturer takes the responsibility of distributor’s inventory levels. Distributor also shares the demand forecast and sales data as well as inventory data. Manufacturer manages the distributor’s inventory with this data. The manufacturer is responsible for determining the order quantities and time in this model [31].

In this study, in cephalosporin department of a factory making secondary pharmaceutical production, a mixed integer mathematical model is developed to obtain the best production plan while maximizing the total net profit in long term. Especially the presence of constraints related to “shelf life, product transition times that are ignored in many studies about pharmaceutical production is also added to the model and an experimental study is implemented. The proposed mathematical model is applied on two different types of supply chain (classical supply chain and vendor managed inventory) and the results of both methods are compared in terms of the total supply chain cost. In addition, the proposed model is also compared with another model from literature to illustrate effectiveness of model.

## Literature review

Planning and scheduling problems have been the subject of innumerable studies in the mathematical programming literature. Various types’ industrial sectors are considered with different time representations in these studies. But some studies cover various industrial sectors for planning and scheduling problem like Fleischmann and Meyr [10]. Pharmaceutical production is a batch process and a type of chemical production. Chemical productions are made in multiproduct plants. The studies for planning and scheduling in multi-product plants are Oh and Karimi [28], Alle and Pinto [1], Dogan and Grossmann [9], Mendez and Cerda [25], Liu et al. [23], Chen et al. [6]. Some studies in literature have handled production and distribution planning together (Lee and Kim [21], Bilgen and Günther [4]).

Also in this part of the study, the studies in the literature related to administrative issues, planning, scheduling and the cost optimization in the pharmaceutical supply chain are listed. The studies about optimization in pharmaceutical supply chain with mathematical methods developed by using mixed integer programming, are as follows; Papageorgiou et al. [29] stduied to optimize the problems in strategic areas such as product development, promotion strategy, capacity planning and investment strategy using a mixed integer programming in the pharmaceutical supply chain. Maravelias and Grossmann [24] discussed simultaneous optimization problem for source constrained scheduling in pharmaceutical production. They proposed MILP that maximizes expected net present value in a multi-period problem. Sundaramoorthy and Karimi [35] developed a MILP including a flexible approach increasing demand meeting ratio against changing production plans in a pharmaceutical supply chain that is the outset of contract manufacturing and new production. Levis and Papageorgiou [22] proposed a mathematical model for long-term capacity planning in a multi-site pharmaceutical industry under uncertainty. The model is an improved version of the model previously proposed by Papageorgiou et al. [29]. All problems are formulated with two-stage MILP model. Then, they developed a hierarchical algorithm for solving large-scale problems. The accuracy of their proposed method was displayed by comparing with several examples. Kim [18] applied an integrated approach to the pharmaceutical supply chain in the health sector. The aim was to reduce holding cost and to optimize inventory costs. For this, VMI was applied to reduce total supply chain cost. Amaroa and Barbosa [2] developed a planning and scheduling approach in the management of reverse flow supply chain applied in a pharmaceutical company. In their study where optimal production plans were obtained, the economical profit of the model was analyzed separately in terms of supply chain operations and customer satisfaction. Lakhdar and Papageorgiou [19] submitted a mathematical programming approach for medium-term production planning under uncertainty in biopharmaceutical manufacturing. Uncertainty in the study is related to with the fermentation concentration ratio. All problems were discussed in the two-stage multi-scenario planning problem and an algorithm was proposed for the problems in larger size. Venditti [39] developed a heuristic algorithm for production planning in the pharmaceutical industry. Baboli et al. [3] studied pharmaceutical supply chain management with two separate approaches as centralized and decentralized using mathematical programming. They reached to the conclusion that the centralized method reduced the cost much more. Sousaa et al. [34] calculated the dynamic resource allocation problem in the pharmaceutical industry with the delivery costs and the different tax rates to maximize the net profit of the company. Susarla and Karimi [37] taken into account the sequence-dependent pattern change, resource using, maintenance schedules and security stocks using the mathematical model with considering integrated planning and resources in the pharmaceutical production facility. Susarla and Karimi [36] studied to optimize supply chain costs about integrated supply chain planning more than one production plant for pharmaceutical production activities. Kelle et al. [17] developed a solution with MILP for demand point in a hospital pharmaceutical supply chain organized medication requirements plan. Chen et al. [7] used a simulation-based optimization technique while increasing customer service level and reducing supply chain costs of pharmaceutical clinical trials and they planned production and distribution activities with MILP. Kelle et al. [17] developed a strategic and tactical level of decision support models in their work related to pharmaceutical supply chain and inventory solutions in a hospital. Kabra et al. [14] planned multi-stage and multi-production processes in the biopharmaceutical production as long-term by using MILP.

The studies about administrative subjects that made extended literature survey in pharmaceutical supply chain can be listed as follows; Shah [32] determined the key issues and optimization strategies for pharmaceutical supply chain in the study to determine the pharmaceutical supply chain and optimization strategies. In the study, by mentioning all pharmaceutical processes, from raw material production until delivered to the customer were explained and made some suggestions about how to increase customer service level. Besides, Shah [32] analyzed all stages one by one ensuring added value to the pharmaceutical supply chain and emphasized the important matters. Yu et al. [40] conducted a study making an evaluation for current issues and health system reform about pharmaceutical supply chain in China. Jaberidoost et al. [13] studied revealing strategic risks of supply chain management in pharmaceutical industry and they mentioned different studies about this area. Narayana et al. [26] discussed the existing studies on the pharmaceutical supply chain in their study. They classified the studies in literature according to the countries, research methods, terminology and the level of analysis. They made evaluations about the future of the studies from the administrative perspective.

In addition the studies about the implementation of VMI model in the pharmaceutical industry are limited. These are; Danese [8] discussed the project of the implementation of VMI to the entire production and distribution facilities and expressed how to manage the entire supply chain with VMI model from one side, data networks established and data systems supporting them and the performances of them in detail. This VMI model as seen in the study was a model applicable to the entire pharmaceutical industry. Shen et al. [33] tried to identify economic production amount under minimum volume constraints in the production of perishable products such as pharmaceuticals that is important for public health. They developed an approach consists of a mathematical model and VMI which was more economic in in terms of total supply chain cost. Kannan et al. [15] proposed a model revealing the benefits of VMI in pharmaceutical industry. Kannan [16] tried to identify the most appropriate supply policy to stochastic demand environment depending on the time with VMI in pharmaceutical industry. All these studies summarized as seen in Table 1.

**Table 1:** Studies about optimization in pharmaceutical industry

The subject of the study	Method	Authors
Planning, scheduling and cost optimization in pharmaceutical industry	Classical supply chain model	Papageorgiou et al. [29], Maravelias and Grossmann [24], Sundaramoorthy and Karimi [35], Levis and Papageorgiou [22], Shah [32], Lakhdar and Papageorgiou [19], Amaroa and Barbosa [2], Venditti [39] Yu et al. [40], Baboli et al. [3], Sousaa et al. [34], Susarla and Karimi [37], Susarla and Karimi [36], Kelle et al. [17], Chen et al. [7], Kabra et al. [14], Jaberidoost et al. [13], Narayana et al. [26]
	Vendor managed inventory model	Danese [8], Shen et al. [33], Kannan et al. [15], Kannan [16]

In this study, a new mathematical model is developed to solve an inventory problem in the pharmaceutical industry. The mathematical model contains shelf life and product transition constraints together and the model is much more dealing with real life constraints unlike the studies in literature. Besides, the model contains parameters about general supply chain parameters such as costs of production, inventory holding, transition, waste product and unmet demand penalty. There are a few articles about VMI method in pharmaceutical sector. The model applied to classical supply chain method and VMI, so this study is implemented VMI method and handled a new technic in pharmaceutical supply chain.

## Methods

### The problem and detailed mathematical formulation

The studies in the literature discuss a simplified model of production occurs in real life. In models, they take into account only materials and machinery as resources and mathematical models try to solve the model by making critical assumptions about the case such as transit or installation times of material transfers, human resources, waste storage and treatment capacity. These assumptions prevent to be applicable of the models established in practice. However, the studies should include more comprehensive models to completely adapt to real life. To make a decision by combining entire supply chain under unique plan in real life is very difficult in dynamic market and environment conditions. Some problems in literature (Shah [32], Yu et al. [40], Jaberidoost et al. [13], Narayana et al. [26]) are studied solution suggestions by taking into account the supply, production and distribution processes. However, simple and flexible models are ensuring rapid solution and being appropriate for the real life are much needed. Although it is generally ignored in literature, pharmaceutical raw material and end product have a definite expiry date and it cannot be used after the expiry date. If there is a presence of raw material and product inventory in warehouse, they are turned into waste product and they will reflect to the supply chain as waste cost. For this reason, the raw materials and product expiry conditions must be taken into account.

In this study, a long-term planning model is developed in order to obtain the production plan that optimally fulfills a net profit objective. This criterion presents the trade between sales returns and costs issues. The main contribution of this study is to solve an inventory problem in the pharmaceutical industry by proposing a new mathematical model that contains shelf life and product transition constraints together. We believe that the proposed model is much more dealing with real life constraints unlike the studies in literature. Besides, the model contains general supply chain parameters such as costs of production, inventory holding, transition, waste product and unmet demand penalty.

A hybrid time representation is applied over a planning horizon, in which the months of the planning horizon are modeled and each month is represented by a continuous time formulation. The most effective characteristic of the problem is that, inventory amounts depend on the shelf life of the products. Also, transition conditions are handled that occur while switching from one product to another.

In the mathematical model, constraints about transitions are adopted from the models of Liu et al. [23]. However, being different from them in our model, inventory amounts changes depending on the shelf life of the product. And so; all inventory formulations are novel. The cost criterion subtracted from the total sales revenue in the objective function is novel in this model. Nomenclature of the proposed mathematical model is given at the Appendix 1. There is a little literature about VMI method in pharmaceutical sector. So this study is implemented VMI method and handled a new technic in pharmaceutical supply chain.

As close as a real life problem, a hypothetical problem is considered in cephalosporin department of a pharmaceutical factory. Our mathematical model is applied in order to obtain the best production plan while maximizing total net profit in long term. The efficiency of the model is shown on classical supply chain and VMI method.

### Objective function

The total net profit is maximized to obtain from sales revenue minus supply chain costs, involving the total, production cost, product transition costs, the unmet demand costs, inventory holding cost, product transportation cost and the cost of waste products.

1 $$ \mathrm{N}\mathrm{P} = {\displaystyle \sum_c}{\displaystyle \sum_i}{\displaystyle \sum_t}P{S}_{ic}*{S}_{cit}-{\displaystyle \sum_i}{\displaystyle \sum_t}{P}_{it}*C{P}_i - {\displaystyle \sum_i}{\displaystyle \sum_{j\ne i}}{\displaystyle \sum_t}C{C}_{ij}*{Z}_{ijt}-{\displaystyle \sum_i}{\displaystyle \sum_{j\ne i}}{\displaystyle \sum_{t\in T-\left\{1\right\}}}C{C}_{ij}*Z{F}_{ijt}-{\displaystyle \sum_c}{\displaystyle \sum_i}{\displaystyle \sum_t}CU{D}_{ic}*U{D}_{cit}-{\displaystyle \sum_i}{\displaystyle \sum_t}C{I}_i*S{M}_{it}-{\displaystyle \sum_c}{\displaystyle \sum_i}{\displaystyle \sum_t}T{C}_i*{S}_{cit}-{\displaystyle \sum_i}{\displaystyle \sum_t}C{W}_i*{W}_{it} $$

### Product assignment constraints

2 $$ {\displaystyle \sum_i}{F}_{it}=1\kern1.75em \forall\ t\in T $$

3 $$ {\displaystyle \sum_i}{L}_{it}=1\kern1.5em \forall\ t\in T $$

In each period one product is assigned as first or last product to be processed and these two equations show whether they are the first product or last product.

4 $$ {F}_{it}\le\ {E}_{it}\kern1.5em \forall\ i\in I,\kern0.75em t\in T $$

5 $$ {L}_{it}\le {E}_{it}\kern1.75em \forall\ i\in I,\kern1em t\in T $$

When the relevant product is not produced in this period, it assumes as E_it_ = 0.

### Product transition constraints

6 $$ {\displaystyle \sum_{i\ne j}}{Z}_{ijt} = {E}_{jt}-{F}_{jt}\kern1em \forall\ i\in I,\ j\in J\kern0.75em t\in T $$

7 $$ {\displaystyle \sum_{j\ne i}}{Z}_{ijt} = {E}_{it}-{L}_{it}\kern0.75em \forall\ i\in I,\ j\in J\kern0.75em t\in T $$

While Z_ijt_ binary variables are representing the product transitions occurring in a period, ZF_ijt_ variable represents the product transition between two consecutive periods.

If there is a product transition within a period, they are the variables indicating that there will be no product before the first product produced and it is not the first product, other products will give priority to this product.

Similarly, if there is a product transition within a period, there will not be any product production after the last product produced and if it is not the last product, this product will be followed by the production of other products.

8 $$ {\displaystyle \sum_i}Z{F}_{ijt}={F}_{jt}\kern1em \forall\ i\in I,\ j\in J\kern0.75em t\in T-\left\{\ 1\right\} $$

9 $$ {\displaystyle \sum_i}Z{F}_{ijt}={L}_{it-1}\kern1em \forall\ i\in I,\ j\in J\kern0.75em t\in T-\left\{\ 1\right\} $$

If there is a product transition between two consecutive periods and if the production of a product begins in that period for the first time, there will certainly be a product transition a period before the relevant period. If a product is not the first or the last one processed, then there is not a changeover involving the product between two periods.

### Travelling salesman problem formulation based subtour prevention constraints

10 $$ {\beta}_{jt}-\left({\beta}_{it}+1\right)\ge -M*\left(1-{Z}_{ijt}\right)\kern1em \forall\ i\in I, \kern0.5em j\in J, \kern0.5em t\in T,\kern1em j\ne i $$

11 $$ {\beta}_{it}\ \le M*{E}_{it}\kern1.25em \forall\ i\in I, \kern0.5em t\in T $$

*β*_*it*_variable can be called as demand index or production row. The aim of writing these constraints is to determine row of the product in a period and to take the product transition cases under control.

If i^th^ product is produced before j^th^ product, production sequence number of i^th^ product will be at least one more than j^th^ product.

If that product has never been produced, demand index will be zero.

These constraints are similar to the constraints preventing the sub-tours in classical Travelling Salesman problem (TSP) [23]. In TSP problem binary variables are used to represent transition one city to another. As similar in this model to maximize net profit there should be minimum number of transition in production sequence. And Z_ijt_ and ZF_ijt_ variables are added to model the product transition in a period and between t-1 and t periods respectively.

12 $$ {F}_{it}\ \le {\beta}_{it}\ \le {\displaystyle \sum_j}{E}_{jt}\kern1.75em \forall\ i\in I, \kern0.5em t\in T $$

This constraint enables the demand index to take at least the value of 1 and to take value up to the maximum product number.

### Timing constraints

13 $$ {\theta}^L\ *{E}_{it}\ \le {O}_{it}\le {\theta}^U\ *{E}_{it\kern0.5em }\kern1.75em \forall\ i\in I, \kern0.5em t\in T $$

For each product produced in a period, the highest and lowest time limits are given.

14 $$ {\displaystyle \sum_i}{O}_{it}+{\displaystyle \sum_i}\left(\left({Z}_{ijt}+Z{F}_{ijt}\right)*T{Z}_{ijt}\right)\le {\theta}^{U\ }\kern2em \forall \kern0.5em t\in T-\left\{1\right\} $$

15 $$ {\displaystyle \sum_i}{O}_{it}+{\displaystyle \sum_i}\left({Z}_{ijt}*T{Z}_{ijt}\right)\le\ {\theta}^{U\ }\kern1.75em \forall \kern0.5em t\in \left\{1\right\} $$

The total of the production in a period and product transition times cannot exceed the existing time given for the shift.

### Production constraints

16 $$ {P}_{it}=r{r}_i*{O}_{it}\kern1.25em \forall\ i\in I, \kern0.5em t\in T $$

The product amount produced in a period is as much as the multiplication of the production ratio and production time.

### Demand constraints

17 $$ \begin{array}{cc}\hfill U{D}_{cit}=R{D}_{cit}-{S}_{cit}\hfill & \hfill\ \forall\ c\in C,\kern0.5em i\in I, \kern0.5em t\in T\hfill \end{array} $$

The amount of the unmet demand from a product is as much as the difference between the realized demand within that period and the product amount delivered to the customer (satisfied demand) within that period.

18 $$ {S}_{cit}\kern0.5em \le R{D}_{cit}\kern1.5em \forall\ c\in C,\kern0.5em i\in I, \kern0.5em t\in T $$

Sales amount may be lower than or equal to demand amount that was realized; in this model the unmet demands in a period are not delivered to the customer in the next period (no backlogs).

### Shelf life and inventory constraints

19 $$ S{M}_{it}=S{M}_{it-1}+{P}_{it}-{\displaystyle \sum_c}{S}_{cit}\kern2.25em  if\kern0.5em t < {\alpha}_i\kern0.75em \forall\ i\in I, t\in T $$

20 $$ S{M}_{it} = {\displaystyle \sum_{t+1-{\alpha}_i}^{t-1}}S{M}_{it-1}+{P}_{it}\kern2.75em  if\kern1em t\ge\ {\alpha}_i\kern2em \forall\ i\in I, t\in T $$

α_i_ is shelf life and depends on products characteristics and it is defined as an integer multiple of t.

Inventory quantity varies depending on the product shelf life. If the relevant time is lower than the product’s shelf life, that is to say, if the product’s term has not been expired yet, the inventory amount of that product is as much as the difference of the sales amount within that period in the total of the stock amount transferred from the previous period and the product amount produced in that period.

21 $$ {W}_{it}={\displaystyle \sum_{t=1}^{t-{\alpha}_i}}S{M}_{it},\kern0.5em  if\kern2em t\ge\ {\alpha}_i\kern1.75em \forall\ i\in I, t\in T $$

But if the relevant time is longer than the product’s shelf life, the products whose term is expired will be waste product.

22 $$ {W}_{it}=0\kern0.5em ,\kern0.5em  if\kern0.75em t < {\alpha}_i $$

### Numerical investigation

Around the proposed mathematical model, our main research focus is addressed in numerical investigation and the following questions are answered:

- Does this mathematical model provide reasonable results for a long term planning horizon?
- Does the proposed mathematical model offer cost advantages?
- How does the proposed model run for different demand profiles and different numbers of products?
- Does this mathematical model run for different kind of supply chain methods?
- Which supply chain method is more profitable with this mathematical model?
- When compared other studies in literature, does this model provide advantage?

To answer these questions, an experimental study is considered based on numerical experiments and comparisons. Experimental set is summarized in Table 2.

**Table 2:** Experimental set

Experiment No	Supply chain method	Planning horizon (months)	Product amounts
1	Classical supply chain	24	10
2	Classical supply chain	24	15
3	Classical supply chain	30	10
4	Classical supply chain	30	15
5	VMI	24	10
6	VMI	24	15
7	VMI	30	10
8	VMI	30	15

To illustrate the applicability of our mathematical model, we consider a hypothetical pharmaceutical plant. In this case, long term production scheduling problem in the pharmaceutical secondary production is discussed.

In the cephalosporin department of this factory, injectable beta-lactam products are manufactured. In a separate facility that is completely independent from non-beta - lactam production fields, micro powder refilling is conducted in aseptic conditions and the production conditions are provided to be monitored continuously via computer aided production systems.

In our study, it is aimed to satisfy the customer demands for 15 different products produced in cephalosporin department of the factory in 24 and 30 months period to find optimal production plan maximizing the total net profit and to show in which type of supply chain (classical or vendor managed).

**Fig. 1:**
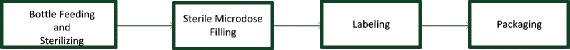
Factory’s transportation system and warehouses

We hypothesized that; the pharmaceutical factory has agreements with 5 pharmaceutical warehouses. In Fig. 1 factory’s transportation system and warehouses scheme is given. The factory delivers the products to pharmaceutical stores and pharmaceutical stores deliver them to the pharmacies. Before the expiry, all the products that are not delivered to the consumer return to the factory and its disposal is carried out by the factory. In this way, it is seen that the production, delivery and waste product costs (disposal cost) belonging to the products whose term is expired and that will be returned being sold, creates serious cost damages to the factory and a mathematical model is developed to minimize these costs and the model is compared by being applied in two separate types of supply chain. In this study, product name is not specified and 15 different products are named according to the active raw materials. These are shown in Table 3.

**Table 3:** Cephalosporin products

No	Active raw material
1	Ceftriaxone 1 g IM/IV
2	Cefotaxime 1 g IM/IV
3	Ceftizoxime 0,5 g IM/IV
4	Cefsulodin 1 g IM/IV
5	Cefoperazone 1 g IM/IV
6	Ceftazidime 0,5 g IM/IV
7	Moxalactam 1 g IM/IV
8	Cefuroxime 0,5 g IM/IV
9	Cephalothin 0,5 g IM/IV
10	Cephapyrine 1 g IM/IV
11	Cefdinir 0,5 g IM/IV
12	Cefprozil 1 g IM/IV
13	Ceftibuten 0,5 g IM/IV
14	Cefpodoxim Proxetil 0,5 g IM/IV
15	Cefaclor Monohydrate 1 g IM/IV

**Fig. 2:**
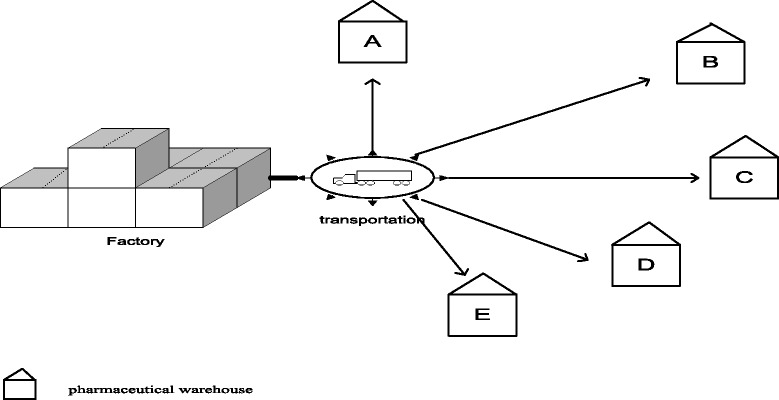
Cephalosporin production area work flow chart

A work flow chart belonging to cephalosporin production site is seen above in the Fig. 2. As shown here, the production is performed on a unique line. Filling machine is isolated from the production environment and in a sterile manner. The active material is loaded into the filling machine; these raw materials are again filled in pharmaceutical bottles in a sterilized way; the bottles filled are checked, labeled and boxed. The production and the expiry date determining the shelf life of the product are printed on the label and box when the product is on the line during relevant transitions.

### Key model parameters

#### Demand types

Demand forecast was made while making production plan for the next two years. Forecasted demand and sales amounts were obtained from the real old data belonging to a real pharmaceutical company. The average of sales amounts (29000) can be seen to conform to normal distribution having standard deviation (10500).

Since normal probability distribution a widely known type, in many studies related to inventory management in supply chain that demand structure is in accordance with normal distribution (Lau et al. [20]).

23 $$ F{D}_{it}= normal\ \left(29000,10500\right) $$

Equation 23 gives the distribution of forecasted demand amounts.

In current and proposed situation, the average of customer demands is 27500 and it is assumed to comply with the normal distribution, the standard deviation of which is 19000. The equation 24, gives the distribution of customer demands performed.

24 $$ R{D}_{it}= normal\ \left(27500,19000\right) $$

#### Production capacity

In literature there are studies in accordance with uniform distributed production capacities [5, 38]. The aim of the manufacturers is always to satisfy the demand completely. Maximum production capacities are limited, but this capacity varies according to the events to occur during the processes (malfunction or periodic maintenances, or even stops).

The factory works 12 h a day. Total available processing time in a month is 264 h. Production capacity is up to maximum 4500 boxes of product/hour. But this capacity vary in real life because of deteriorations or stops in production lines. When it is statistically analyzed, the capacity of cephalosporin department production line is uniformly distributed in the range of (0, 4500).

#### Shelf life conditions

The shelf life of each product is fixed and it is 12 months. The products whose terms are expired cannot sold within this time period, it is assumed as waste products and the costs belonging to this reflect as waste product cost (ie. 0.125 $/unit).

#### Product transition conditions

Transition cost is proportional to transition times by a factor of 10.

Unit inventory holding cost data are the values such as 0.005 $/unit identified by the company for a package of product, unit sales price and rest of the other costs are given in Table 4.

**Table 4:** Unit costs and sales price data of products

Product no	Sales price ($)	Production cost ($)	Unmet demand cost ($)	Transition cost ($)
Product 1	7	1,2	0,2	TZij *10
Product 2	7	1,2	0,25	TZij *10
Product 3	7	1,16	0,18	TZij *10
Product 4	7	1,16	0,25	TZij *10
Product 5	7	1,2	0,2	TZij *10
Product 6	7	1,1	0,15	TZij *10
Product 7	7	1	0,15	TZij *10
Product 8	10	1,25	0,2	TZij *10
Product 9	10	1,2	0,18	TZij *10
Product 10	10	1,2	0,22	TZij *10
Product 11	10	1,16	0,25	TZij *10
Product 12	8	1,2	0,22	TZij *10
Product 13	8	1,16	0,25	TZij *10
Product 14	8	1,16	0,15	TZij *10
Product 15	8	1,2	0,22	TZij *10

Product transportation costs are varies to warehouses and given in Table 5.

**Table 5:** Unit product transportation costs to warehouses

Pharmaceutical warehouses	Transportation costs for unit product
A	0,005
B	0,02
C	0,017
D	0,015
E	0,045

The transition times from one product to another (cleaning and mold change) are variable and they are stated in Table 6 below.

**Table 6:** The transition time (hour) from i to j

i\j	1	2	3	4	5	6	7	8	9	10	11	12	13	14	15
1	0	10	7	8	7	9	8	12	10	8	7	6	9	7	10
2	12	0	10	6	12	9	7	8	8	12	9	8	8	8	9
3	8	6	0	7	11	8	10	8	9	6	8	9	7	8	8
4	8	9	7	0	12	10	8	9	9	6	7	10	8	9	6
5	6	12	8	12	0	7	11	14	7	9	6	7	11	8	10
6	14	10	6	14	12	0	10	9	12	6	9	9	7	7	9
7	10	8	11	8	9	7	0	8	7	9	8	11	7	9	8
8	7	11	7	9	12	6	8	0	9	7	8	10	9	12	6
9	9	12	8	12	12	8	7	9	0	10	9	8	7	9	8
10	6	8	10	11	7	9	8	8	10	0	6	9	8	10	8
11	11	6	9	7	6	11	8	10	6	9	0	11	7	8	10
12	8	11	8	9	7	8	6	11	7	9	8	0	6	11	7
13	10	8	7	9	8	11	8	9	10	7	8	10	0	8	9
14	9	7	9	12	8	12	12	8	7	9	8	9	8	0	7
15	8	7	9	8	7	11	8	10	9	6	11	8	10	7	0

## Results

### Details of experiments

In this part of study, some of the experimental results are given for comparison. Firstly Experiment Number 1 and 5’s details are given and compared.

**Fig. 3:**
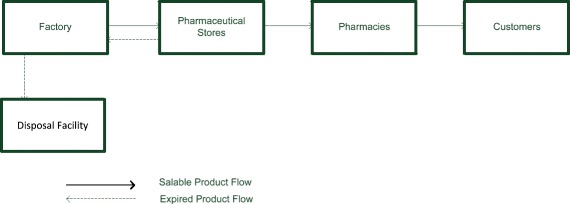
Pharmaceutical supply chain processes

In Experiment 1; factory has a classical supply chain model and produced products transfer due to the orders from pharmaceutical stores and deliver to the end user from there. The products which are expired before sold from the factory to the pharmaceutical store are sent to disposal facility. Besides, the products which is expired in the pharmaceutical store before sold to pharmacy, are sent to the producer company by the pharmaceutical stores and when these products are reached to the producer company, it is taken to “reject store” and it is delivered to the company for disposal facilities. During this returning process, all costs (transport + disposal costs) are subject to the company. A scheme of pharmaceutical supply chain processes can be seen in Fig. 3.

**Fig. 4:**
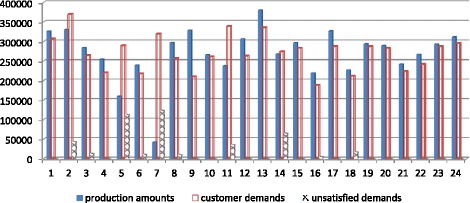
The graph of production, demand and the unsatisfied demand for Experiment 1

The above example is modeled using GAMS/CPLEX 12 for the MILP optimization. When the model is run, maximized total net profit is 42556741$ for 24 months with 10 products. The graphs including demand amounts, production amounts, stock amounts and unmet demand amounts belonging to the model are given in following Fig. 4.

**Fig. 5:**
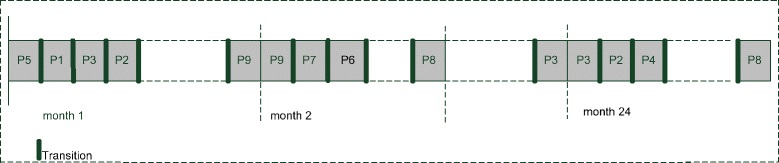
Production schedule for Experiment 1

Production schedule is given in Fig. 5. This figure shows how the transition times affects to the production sequence. It is hard to show all planning horizon (24 months) scheduling, so we illustrate for a part of two months planning time.

In Experiment 5, the inventory is vendor-managed. According to the agreement, pharmaceutical factory can see the inventory and sales information in the pharmaceutical stores online with the established information system. At the beginning, a production plan is created according to estimated demand amounts and with the information obtained from pharmaceutical store, production plans are updated and within the framework of these plans, delivery plans will be made and the product will be delivered. The amounts and time information will be communicated with the store. For each product, the level of safety stocks in the store is determined and the stocks will be controlled continuously in order not to fall under safety stock levels in the store. Safety stock levels are given in Table 7. Equation 16 in Exp. 1 turns into the equation 25 in Exp.5.

**Table 7:** Safety stock levels

Product	Product active item	Safety stock amount
Product 1	Ceftriaxone 1 g IM/IV	1000
Product 2	Cefotaxime 1 g IM/IV	2250
Product 3	Ceftizoxime 0.5 g IM/IV	350
Product 4	Cefsulodin 1 g IM/IV	500
Product 5	Cefoperazone 1 g IM/IV	175
Product 6	Ceftazidime 0.5 g IM/IV	1250
Product 7	Moxalactam 1 g IM/IV	350
Product 8	Cefuroxime 0.5 g IM/IV	175
Product 9	Cephalothin 0.5 g IM/IV	1450
Product 10	Cephapyrine 1 g IM/IV	1250
Product 11	Cefdinir 0,5 g IM/IV	250
Product 12	Cefprozil 1 g IM/IV	1000
Product 13	Ceftibuten 0,5 g IM/IV	750
Product 14	Cefpodoxim Proxetil 0,5 g IM/IV	700
Product 15	Cefaclor Monohydrate 1 g IM/IV	350

25 $$ {P}_{it}=F{D}_{cit}+S{S}_{\dot{I}}-S{M}_{it-1}\kern1.25em \forall\ i\in I, t\in T $$

26 $$ \begin{array}{cc}\hfill {S}_{cit}\kern0.5em ={P}_{it}\hfill & \hfill \forall\ c\in C,\ i\in I, t\in T\hfill \end{array} $$

In Experiment 5, each product is sold to pharmaceutical stores according to the agreement. For this reason, in addition to the equation 18 created for sales amounts, the equation 26 above should be added. According to production plans, the products produced in the factory are directly sent to the pharmaceutical store. That is to say, sales amount is equal to the production amount. Then, the production plans are revised from month to month in the factory in accordance with the demands. Here, the aim is to prevent shortages and waste product costs and making the supply chain more profitable.

**Fig. 6:**
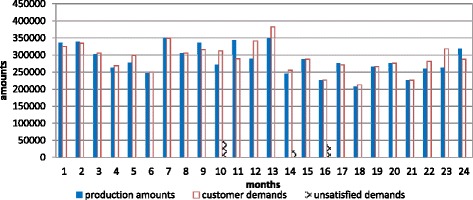
The graph of production, demand and the unmet demand amounts for Experiment 5

When the model is run, the maximized total net profit is 47389739 $ for 24 months with 10 products. The graphs including demand amounts, production amounts, stock amounts and unmet demand belonging to the Experiment 5 are given in Fig. 6.

The result of comparison for these two experiments is as follows in Table 8.

**Table 8:** Comparison of solution results for experiment 1 and 5 for 24 months with 10 products

		Exp. 1	Exp. 5
Revenue ($)			
	*Sales revenue*	50823262	55825164
Costs ($)			
	*Production cost*	8131853	8412132
	*Transition cost*	5030	4050
	*Unmet demand cost*	78908	4012
	*Inventory holding cost*	29963	7876
	*Product transportation cost*	6612	7028
	*Disposal cost*	14153	325
Total net profit ($)		42556741	47389739

**Fig. 7:**
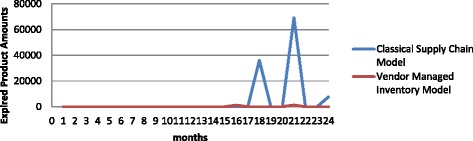
Expired product amounts for Experiment 1 and 5

The mathematical model created to reveal the best production plan while minimizing supply chain costs and maximizing the total net profit. The model compared with two experiments. For the both situations, the common data and data distributions are used. Two different optimum results are obtained according to supply chain types. When these results are compared, sales revenue in Experiment 1 is less than in Experiment 5. The sales revenue difference between them is so much. Transition costs are also seen as more advantageous in Experiment 5. The unmet demand amounts are less in Experiment 5 and accordingly, its cost is lower. Production and transportation costs in Experiment 1 is less than Experiment 5. This is because the sales amounts are less than Experiment 5. Considering waste product costs, in Experiment 1, 1.72 % of the production is waste product and in Experiment 5, this rate is 0.03 %. So we can say, VMI is more advantageous in terms of waste product cost and wastage amounts. Because, the production plans advance in more controlled way in Experiment 5. A comparison of these experiments’ expired product amounts can be seen in Fig. 7. When total net profit is compared, VMI is more profitable as much as 10.19 % than Classical Supply Chain. Finally, when two experiments are compared, VMI is much better in all costs except for production and transportation costs.

The model is run for all experimental set and the total net profit for all the experiments are given below in Table 9.

**Table 9:** Solution results for all experimental set

Experiment no	Supply chain method	Planning horizon (months)	Product amounts	Total net profit ($)
1	Classical supply chain	24	10	42556741
2	Classical supply chain	24	15	61625811
3	Classical supply chain	30	10	52489164
4	Classical supply chain	30	15	72209637
5	VMI	24	10	47389739
6	VMI	24	15	66800579
7	VMI	30	10	55163846
8	VMI	30	15	79625469

As seen in Table 8, VMI Method is more profitable (nearly 8,8 %) than Classical Supply Chain method.

Production planning is a crucial issue in pharmaceutical supply chain in terms of meeting customer demands just in time. Because pharmaceuticals are perishable products, the shelf life constraints have to be considered while planning, scheduling and all supply chain activities are organized.

According to our limited knowledge, some pharmaceutical companies do not take into account the shelf life constraints during their planning processes. It is assumed that all produced items will be sold after reasonable waiting time in the inventory. In reality, in some cases, there can be long waiting time of products. So their perishing day of products can be very close to shelf life because of long waiting time. Although the long waited products are still considered as “inventory products” from the planner, they must be considered “wasted” instead of inventory product.

In this study we consider shelf life constraints and product transition constraints together. In terms of the practical implications of this work in real pharmaceutical companies; the proposed model can be adapted to production planning activities in the pharmaceutical supply chain. Because; the model produces real time inventory information through the shelf life constraints and deals with “long waited (waste) and “inventory” products while the planning is done. This situation has a great opportunity to cope with wastage costs. The model balances the inventory levels, demands and lost sales. And also real time inventory information provides to reduce risks about market demands and efficient production scheduling activities. This helps to reduce all supply chain costs and the model offers a collaborative, planning and scheduling system to pharmaceutical companies while managing shelf life of products.

## Discussion

### Comparison with another model

In this part of study, the efficiency of our model is compared with Chen et al. [6] model. To make a comparison, the Chen et al. [6] mathematical model is modified. Modified and original model are given in Appendix 2 and Appendix 3 respectively.

The objective function of proposed model consists of production cost, product delivery cost and wastage cost and it is written as follow:

$$ \mathrm{N}\mathrm{P} = {\displaystyle \sum_c}{\displaystyle \sum_i}{\displaystyle \sum_t}P{S}_{ic}*{S}_{cit}-{\displaystyle \sum_i}{\displaystyle \sum_t}{P}_{it}*C{P}_i - {\displaystyle \sum_i}{\displaystyle \sum_{j\ne i}}{\displaystyle \sum_t}C{C}_{ij}*{Z}_{ijt}-{\displaystyle \sum_c}{\displaystyle \sum_i}{\displaystyle \sum_t}CU{D}_{ic}*U{D}_{cit}-\kern2.5em {\displaystyle \sum_i}{\displaystyle \sum_t}C{I}_i*S{M}_{it}-{\displaystyle \sum_c}{\displaystyle \sum_i}{\displaystyle \sum_t}T{C}_{ic}*{S}_{cit}-{\displaystyle \sum_i}{\displaystyle \sum_t}C{W}_i*{W}_{it} $$

There is no storage capacity limit, so the (C12.) constraint is removed from the model.

In addition, backlogs are not allowed, therefore we remove the constraint (C14) from the model. But we have to calculate unmet demand amount so a new constraint is added as given below:

$$ U{D}_{cit}=R{D}_{cit}-{S}_{cit} $$

The stock amounts is written with considering shelf life conditions, the constraint (C13) as follow:

$$ S{M}_{it}=S{M}_{it-1}+{P}_{it}-{\displaystyle \sum_c}{S}_{cit}\kern2em  if\kern0.5em t < {\alpha}_i\kern0.75em \forall\ i\in I, t\in T $$

$$ S{M}_{it} = {\displaystyle \sum_{t+1-{\alpha}_i}^{t-1}}S{M}_{it-1}+{P}_{it}\kern2.75em  if\kern1em t\ge\ {\alpha}_i\kern2em \forall\ i\in I, t\in T $$

Wastage cost is added to the model;

$$ {W}_{it}={\displaystyle \sum_{t=1}^{t-{\alpha}_i}}S{M}_{it},\kern0.5em  if\kern2em t\ge\ {\alpha}_i\kern1.75em \forall\ i\in I, t\in T $$

$$ \begin{array}{cc}\hfill {W}_{it}=0\kern0.5em ,\hfill & \hfill if\kern0.75em t < {\alpha}_i\hfill \end{array} $$

The model is run under 24 and 30 months production period, the CPU times were 856 and 4150 respectively.

The details of comparison of these models are given in Table 10.

**Table 10:** Comparison results for 24 months and 30 months

		24 moths		30 moths	
		Chen et.al.	The proposed	Chen et.al.	The proposed
Revenue ($)					
	*Sales revenue*	50313184	50823262	62491581	62888928
Costs ($)					
	*Production cost*	8118125	8131853	10172676	10308595
	*Transition cost*	4976	5030	6038	6325
	*Unmet demand cost*	67059	78908	8624	13653
	*Inventory holding cost*	30056,3	29963	36570	42703
	*Product transportation cost*	4590	6612	6038	8145
	*Disposal cost*	10048	14153	17560	20342
Total net profit ($)		42078328	42556741	52244073	52489164

Models were run for 24 and 30 months under classical supply chain. Sales revenues and inventory holding costs are more profitable in our model. And the total net profits are in average %0,8 much more in our model.

## Conclusion

In this study, a new mathematical model was developed to solve inventory problem and maximizing total net profit while determining optimal production plan under shelf life and product transition constraints in the pharmaceutical industry. The proposed MILP model contains “shelf life and product transition times” constraints together and we believe that the proposed model has much more real life constraints unlike the other studies in literature. Besides, the model contains parameters such as costs of production, inventory holding, transition, transport, waste product and unmet demand penalty.

As we defined at literature review section of this study; VMI method is not often used in the literature of the pharmaceutical sector. So this study implemented VMI method and handled a new technic for the pharmaceutical supply chain.

To show the effectiveness of the model, an experimental study; which contains 2 different supply chain policy (Classical and VMI), 24 and 30 months planning horizon, 10 and 15 different cephalosporin products were chosen. The results illustrated that the VMI provided much better results in terms of total supply chain costs. Especially; the waste amount that was very important in pharmaceutical sector and the cost was reduced in the VMI. The waste product amounts were 1.72 and 0.03 % of the products in experiment 1 and experiment 5 respectively. In terms of total supply chain costs, 10.19 % of an advantage was gained by the proposed VMI model. As a result, we believe that the proposed model should be adapted in the pharmaceutical industry to reduce total supply chain cost. In addition that, the proposed model is compared with a recently published study in literature. And so the results are illustrated that the proposed model is better than the other.

## Appendix 1

### Nomenclature

#### Indices

i,j, products

c, customers

t, period

#### Sets

I, J, product set

C, customer set

T, periods set

#### Parameters

CP_i_, unit production cost of i product

CUD_ic_, unit unmet demand penalty cost of product i to customer c

CC_ij_, product transition from product i to product j. (change and cleaning cost on the line while passing from product i to product j)

CI_i_, unit inventory holding cost of product i

CW_i_, unit waste product cost of product i

TC_ic_, unit product delivery cost of product i to customer c

FD_cit_, forecasted demand of customer c, from i th product, at period t

RD_cit_, the demand amount realized by customer c, from i th product, at period t

M, large number

PS_ic_, unit selling price product i to customer c

α_i_, shelf life

Ө^L^, the lowest production rate in a period

Ө^U^, the highest production rate in a period

TZ_i,j_, cleaning and mold changing time while passing from i product to j product

β_it_, demand index

rr_i_, production rate

#### Variables

E_it_ = 1, 0, product i produced in t period?

F_it_ = 1, 0, product i the first product in t period?

L_it_ = 1, 0, product i the last product in period t?

Z_ijt_ = 1, 0, product i produced before product j?

ZF_ijt_ =1,0 if there is any product transition between t-1 and t period?

P_it_, the production amount from product i in period t

S_cit_, sales amount from product i to customer c in period t

O_it_, processing time for product i in period t

SM_it_, inventory amount of product i in period t

SS_i_, security stock amount of product i

UD_cit_, unmet demand amount from product i to customer c in period t

*W*_*it*_, waste product amount product i in period t

NP, total net profit

## Appendix 2

### Modified Chen et. al. model

$$ \mathrm{N}\mathrm{P} = {\displaystyle \sum_c}{\displaystyle \sum_i}{\displaystyle \sum_t}P{S}_{ic}*{S}_{cit}-{\displaystyle \sum_i}{\displaystyle \sum_t}{P}_{it}*C{P}_i - {\displaystyle \sum_i}{\displaystyle \sum_{j\ne i}}{\displaystyle \sum_t}C{C}_{ij}*{Z}_{ijt}-{\displaystyle \sum_c}{\displaystyle \sum_i}{\displaystyle \sum_t}CU{D}_{ic}*U{D}_{cit}-\kern.5em {\displaystyle \sum_i}{\displaystyle \sum_t}C{I}_i*S{M}_{it}-{\displaystyle \sum_c}{\displaystyle \sum_i}{\displaystyle \sum_t}T{C}_{ic}*{S}_{cit}-{\displaystyle \sum_i}{\displaystyle \sum_t}C{W}_i*{W}_{it} $$

$$ {\displaystyle \sum_i}{y}_{it}=1 $$

$$ 0\le {O}_{it}\le {\theta}^U\ *{y}_{it} $$

$$ {\displaystyle \sum_i}{O}_{it}\ge {\theta}^L\ *{y}_{it} $$

$$ {\displaystyle \sum_i}{O}_{it}+\left({\displaystyle \sum_j}T{Z}_{ji}*{Z}_{jit}\right)\le {\theta}^U $$

$$ {\displaystyle \sum_j}{Z}_{ijt}={y}_{it-1\kern0.5em }\kern1.5em t\ne 1 $$

$$ {\displaystyle \sum_i}{Z}_{ijt}={y}_{jt\kern0.5em }\kern0.75em t\ne 1 $$

$$ {\displaystyle \sum_j}{Z}_{ijt+1}={y}_{it} $$

$$ {\displaystyle \sum_i}{Z}_{ijt+1}={y}_{it+1} $$

$$ {P}_{it}=r{r}_i*{O}_{it} $$

$$ S{M}_{it}=S{M}_{it-1}+{P}_{it}-{\displaystyle \sum_c}{S}_{cit}\kern2em  if\kern0.5em t < {\alpha}_i\kern0.75em \forall\ i\in I, t\in T $$

$$ U{D}_{cit}=R{D}_{cit}-{S}_{cit} $$

$$ {S}_{cit}\le R{D}_{cit} $$

$$ S{M}_{it} = {\displaystyle \sum_{t+1-{\alpha}_i}^{t-1}}S{M}_{it-1}+{P}_{it}\kern2.75em  if\kern1em t\ge\ {\alpha}_i\kern2em \forall\ i\in I, t\in T $$

$$ {W}_{it}={\displaystyle \sum_{t=1}^{t-{\alpha}_i}}S{M}_{it},\kern0.5em  if\kern2em t\ge\ {\alpha}_i\kern1.75em \forall\ i\in I, t\in T $$

$$ {W}_{it}=0\kern0.5em ,\kern0.5em  if\kern0.75em t < {\alpha}_i $$

## Appendix 3

The proposed model by Chen et. Al.

### Nomenclature

#### Indices

c, customers

i,j, products

k, time slots

w, weeks

#### Sets

C, customers

I,J, products

Kw, time slots in week w

W, weeks

#### Parameters

CBci, backlog cost of product i to customer c

CIiw, inventory cost of product i in week w

CTij, transition cost of product i to product j

Dciw, demand of product i from customer c in week w

PSci, price of product i to customer c

ri, processing rate of product i

Vimax, maximum storage of product i

Vimin, minimum storage of product i

ӨL, lower bound for processing time

ӨU, upper bound for processing time

τij, changeover time from product i to product j

#### Variables

Pro, operating profit

Piw, production of product i in week w

Sciw, sales of product i to customer c in week w

Tkw, end time of slot k in week w

Viw, volume of product i in week w

Δciw, backlog of product i for customer c in week w

θikw, processing time of product i in slot during week w

#### Binary variables

Eiw, 1 if product i is produced in week w, 0 otherwise

yikw, 1 if product i is produced in time slot k during week w, 0 otherwise

Zijkw, 1 if product i (slot k-1) preceds product j (slot k) in week w, 0 otherwise

### Mathematical formulation

#### Objective function

C1 $$ \mathrm{Pro}={\displaystyle \sum_i}{\displaystyle \sum_w}\left[{\displaystyle \sum_c}\left(P{S}_{ic}*{S}_{ciw}-C{B}_{ic}*{\varDelta}_{ciw}\right)-\left({\displaystyle \sum_j}{\displaystyle \sum_k}C{T}_{ij}*{Z}_{ijkw}+C{I}_{iw}*{V}_{iw}\right)\right] $$

#### Assignment constraints

C2 $$ \begin{array}{cc}\hfill {\displaystyle \sum_i}{y}_{ikw}=1\hfill & \hfill w\in \kern0.5em \mathrm{W}\hfill \end{array} $$

#### Timing constraints

C3 $$ {T}_{0w}=0\kern1.5em w\in \kern0.5em \mathrm{W} $$

C4 $$ 0\le {\theta}_{ikw}\ \le {\theta}^U\ *{y}_{ikw}\kern5.25em i\in \kern0.5em I\kern0.75em k\in K\kern1em w\ \in\ W $$

C5 $$ {\displaystyle \sum_k}{\theta}_{ikw}\kern0.5em \ge {\theta}^L\ *{E}_{iw}\kern1.5em i\in \kern0.5em I\kern0.75em k\in K\kern1em w\ \in\ W $$

C6 $$ {T}_{kw}-{T}_{k-1\ w}={\displaystyle \sum_i}\left({\theta}_{ikw}\kern0.5em +{\displaystyle \sum_i}{\tau}_{ji}*{Z}_{ijkw}\right)\kern1.25em \forall k\in K\kern1em w\ \in\ W $$

#### Transition constraints

C7 $$ {\displaystyle \sum_j}{Z}_{ijkw}={y}_{ik-1w}\kern0.5em i\in \kern0.5em I\kern0.75em k\in K-\left\{1\right\}\kern1em w\ \in\ W $$

C8 $$ {\displaystyle \sum_j}{Z}_{ijkw}={y}_{jkw}\kern0.5em j\in \kern0.5em J\kern0.75em k\in K-\left\{1\right\}\kern1em w\ \in\ W $$

C9 $$ {\displaystyle \sum_j}{Z}_{ij1w+1}={y}_{jKw}\kern0.5em i\in I\kern1.5em w\ \in\ W $$

C10 $$ {\displaystyle \sum_i}{Z}_{ij1w+1}={y}_{j1w+1}\kern1em j\in \kern0.5em J\kern1.5em w\ \in\ W $$

#### Process and storage capacity constraints

C11 $$ {P}_{iw}={r}_i*{\displaystyle \sum_k}{\theta}_{ikw\kern0.5em }i\in I\kern1.5em w\ \in\ W $$

C12 $$ {V}_i^{min}\le {V}_{iw}\ \le {V}_i^{max}\kern0.75em i\in I\kern1.5em w\ \in\ W $$

C13 $$ {V}_{iw} = {V}_{iw+1}\kern0.5em +{P}_{iw}-{\displaystyle \sum_c}{S}_{ciw} $$

C14 $$ {\varDelta}_{ciw}={\varDelta}_{ciw-1}+{D}_{ciw}-{S}_{ciw}\kern1.75em c\ \in C\kern1.5em i\ \in I\kern1.5em w\ \in\ W $$

#### Degeneracy prevention constraints

C15 $$ {\displaystyle \sum_k}{y}_{ikw}\le {E}_{iw} + \left({K}_w\kern0.5em -1\right)*{y}_{iKw}\kern0.75em i\ \in I\kern1.5em w\ \in\ W $$

C16 $$ {E}_{iw}\ \ge {y}_{iKw}\kern0.75em i\ \in I\kern1.5em w\ \in\ W $$

C17 $$ {\displaystyle \sum_{j\ne i}}{\displaystyle \sum_k}\left({Z}_{ijkw}+{Z}_{jikw}\right)\le 2-{y}_{iKw}\kern1.5em i\ \in I\kern1.5em w\ \in\ W $$

## References

[1] Alle A, Pinto JM (2002). Mixed-integer programming models for the scheduling and operational optimization of multiproduct continuous plants. Ind Eng Chem Res.

[2] Amaroa ACS, Barbosa APD (2008). Planning and scheduling of industrial supply chains with reverse flows: a real pharmaceutical case study. Comput Chem Eng.

[3] Baboli A, Fondrevelle J, Moghaddam R, Mehrabi A (2011). A replenishment policy based on joint optimization in a downstream pharmaceutical supply chain: centralized vs. decentralized replenishment. Int J Adv Manuf Technol.

[4] Bilgen B, Günther HO (2010). Integrated production and distribution planning in the fast moving consumer goods industry: a block planning application. OR Spectrum.

[5] Chandra P, Fisher ML (1994). Coordination of production and distribution planning. Eur J Oper Res.

[6] Chen P, Papageorgiou LG, Pinto JM (2008). Medium-term planning of single-stage single-unit multiproduct plants using a hybrid discrete/continuous-time MILP model. Ind Eng Chem Res.

[7] Chen Y, Mockus L, Orcun S, Reklaitis GV (2012). Simulation-optimization approach to clinical trial supply chain management with demand scenario forecast. Comput Chem Eng.

[8] Danese P (2004). Beyond vendor managed inventory: the GlaxoSmithKline case. Supply Chain Forum Int J.

[9] Dogan ME, Grossmann IE (2006). A decomposition method for the simultaneous planning and scheduling of single-stage continuous muliproduct plants. Ind Eng Chem Res.

[10] Fleischmann B, Meyr H (1997). The general lotsizing and scheduling problem. OR Spektrum.

[11] Gaither N (1996). Productions and operations management.

[12] Hines P, Lamming R, Jones D, Cousins P, Rich N (2000). Value stream management- strategy and excellence in the supply chain.

[13] Jaberidoost M, Nikfar S, Abdollahias A, Dinarvand R (2013). Pharmaceutical supply chain risks: a systematic review. DARU J Pharm Sci.

[14] Kabra S, Shaik MA, Rathore AS (2013). Multi-period scheduling of a multi-stage multi-product bio-pharmaceutical process. Comput Chem Eng.

[15] Kannan G, Grigore MC, Devika K, Senthilkumar A (2013). An analysis of the general benefits of a centralised VMI system based on the EOQ model. Int J Prod Res.

[16] Kannan G (2015). The optimal replenishment policy for time-varying stochastic demand under vendor managed inventory. Eur J Oper Res.

[17] Kelle P, Woosleyb J, Schneider H (2012). Pharmaceutical supply chain specifics and inventory solutions for a hospital case. Oper Res Health Care.

[18] Kim D (2005). An integrated supply chain management system: a case study in healthcare sector. E-Commerce Web Technol Proc Lect Notes Comput Sci.

[19] Lakhdar K, Papageorgiou LG (2008). An iterative mixed integer optimisation approach for medium term planning of biopharmaceutical manufacture under uncertainty. Chem Eng Res Des.

[20] Lau JSK, Huang GQ, Mak KL (2004). Impact of information sharing on inventory replenishment in divergent supply chains. Int J Prod Res.

[21] Lee H, Kim H (2000). Optimal production-distribution planning in supply chain management using a hybrid simulation-analytic approach. Proceedings of the 2000 winter simulation conference.

[22] Levis AA, Papageorgiou LG (2004). A hierarchical solution approach for multi-site capacity planning under uncertainty in the pharmaceutical industry. Comput Chem Eng.

[23] Liu S, Pinto JM, Papageorgiou LG (2008). A TSP-based MILP model for medium-term planning of single-stage continuous multiproduct plants. Ind Eng Chem Res.

[24] Maravelias CT, Grossmann IE (2001). Simultaneous planning for new product development and batch manufacturing facilities. Ind Eng Chem Res.

[25] Mendez CA, Cerda J (2002). An efficient MILP continuous-time formulation for short-term scheduling of multiproduct continuous facilities. Comput Chem Eng.

[26] Narayana SA, Pati RK, Vrat P (2014). Managerial research on the pharmaceutical supply chain – a critical review and some insights for future directions. J Purch Supply Manag.

[27] Norman G (1996). Production and operations management.

[28] Oh HC, Karimi IA (2001). Planning production on a single processor with sequence-dependent setups part 1: determination of campaigns. Comput Chem Eng.

[29] Papageorgiou LG, Rotstein GE, Shah N (2001). Strategic supply chain optimization for the pharmaceutical industries. Ind Eng Chem Res.

[30] Ru J (2010). The Impacts of vendor managed inventory on supply chain performance in retail industry.

[31] Salzarulo PA (2006). Vendor managed inventory programs and their effect on supply chain performance.

[32] Shah N (2004). Pharmaceutical supply chains: key issues and strategies for optimisation. Comput Chem Eng.

[33] Shen Z, Dessouky M, Ordonez F (2011). Perishable inventory management system with a minimum volume constraint. J Oper Res Soc.

[34] Sousaa RT, Liu S, Papageorgiou LG, Shah N (2011). Global supply chain planning for pharmaceuticals. Chem Eng Res Des.

[35] Sundaramoorthy A, Karimi IA (2004). Planning in pharmaceutical supply chains with outsourcing and new product introductions. Ind Eng Chem Res.

[36] Susarla N, Karimi IA (2012). Integrated supply chain planning for multinational pharmaceutical enterprises. Comput Chem Eng.

[37] Susarla N, Karimi IA (2011). Integrated campaign planning and resource allocation in batch plants. Comput Chem Eng.

[38] Tosto GD, Parunak VD (2009). Multi-agent-based simulation X: international workshop.

[39] Venditti L (2010). Production scheduling in pharmaceutical industry. PhD dissertation.

[40] Yu X, Li C, Shi Y, Yu M (2010). Pharmaceutical supply chain in China: current issues and implications for health system reform. Health Policy.

